# Epigenetic Mechanisms Regulate MHC and Antigen Processing Molecules in Human Embryonic and Induced Pluripotent Stem Cells

**DOI:** 10.1371/journal.pone.0010192

**Published:** 2010-04-16

**Authors:** Beatriz Suárez-Álvarez, Ramón M. Rodriguez, Vincenzo Calvanese, Miguel A. Blanco-Gelaz, Steve T. Suhr, Francisco Ortega, Jesus Otero, Jose B. Cibelli, Harry Moore, Mario F. Fraga, Carlos López-Larrea

**Affiliations:** 1 Histocompatibility and Transplantation Unit, Hospital Universitario Central de Asturias, Oviedo, Spain; 2 Department of Animal Science and Physiology, Michigan State University, East Lansing, Michigan, United States of America; 3 Department of Nephrology, Hospital Universitario Central de Asturias, Oviedo, Spain; 4 Unidad de Coordinación de Trasplantes y Terapia Celular, Hospital Universitario Central de Asturias, Oviedo, Spain; 5 Cancer Epigenetics Laboratory, Instituto Universitario de Oncología del Principado de Asturias (IUOPA), Hospital Universitario Central de Asturias, Oviedo, Spain; 6 Department of Immunology and Oncology, National Center for Biotechnology, CNB-CSIC, Cantoblanco, Madrid, Spain; 7 Programa Andaluz de Terapia Celular y Medicina Regenerativa, Andalucía, Spain; 8 Centre for Stem Cell Biology, University of Sheffield, Western Bank, Sheffield, United Kingdom; 9 Fundación Renal “Íñigo Álvarez de Toledo”, Madrid, Spain; Brunel University, United Kingdom

## Abstract

**Background:**

Human embryonic stem cells (hESCs) are an attractive resource for new therapeutic approaches that involve tissue regeneration. hESCs have exhibited low immunogenicity due to low levels of Mayor Histocompatibility Complex (MHC) class-I and absence of MHC class-II expression. Nevertheless, the mechanisms regulating MHC expression in hESCs had not been explored.

**Methodology/Principal Findings:**

We analyzed the expression levels of classical and non-classical MHC class-I, MHC class-II molecules, antigen-processing machinery (APM) components and NKG2D ligands (NKG2D-L) in hESCs, induced pluripotent stem cells (iPSCs) and NTera2 (NT2) teratocarcinoma cell line. Epigenetic mechanisms involved in the regulation of these genes were investigated by bisulfite sequencing and chromatin immunoprecipitation (ChIP) assays. We showed that low levels of MHC class-I molecules were associated with absent or reduced expression of the transporter associated with antigen processing 1 (TAP-1) and tapasin (TPN) components in hESCs and iPSCs, which are involved in the transport and load of peptides. Furthermore, lack of β2-microglobulin (β2m) light chain in these cells limited the expression of MHC class I trimeric molecule on the cell surface. NKG2D ligands (MICA, MICB) were observed in all pluripotent stem cells lines. Epigenetic analysis showed that H3K9me3 repressed the TPN gene in undifferentiated cells whilst HLA-B and β2m acquired the H3K4me3 modification during the differentiation to embryoid bodies (EBs). Absence of HLA-DR and HLA-G expression was regulated by DNA methylation.

**Conclusions/Significance:**

Our data provide fundamental evidence for the epigenetic control of MHC in hESCs and iPSCs. Reduced MHC class I and class II expression in hESCs and iPSCs can limit their recognition by the immune response against these cells. The knowledge of these mechanisms will further allow the development of strategies to induce tolerance and improve stem cell allograft acceptance.

## Introduction

Human embryonic stem cells (hESCs) are pluripotent cells derived from the inner cell mass of blastocysts. hESCs have the capacity to differentiate into all tissues of the body, making them useful in regenerative medicine. Nevertheless, elucidation of the immunogenicity of hESCs-derived allografts, and their potential rejection by the recipient remains elusive. Major histocompatibility complex (MHC) class I antigen processing and presentation is required for effective T cell recognition and impacts graft rejection. Early work showed that hESCs express very low levels of MHC class I molecules on the cell surface and fail to elicit immune responses in immune-competent mice [1], supporting the hypothesis that these cells have immune-privilege properties which expands their use in cell replacement therapy [2]–[5]. Several reproductive and developmental tissues such as sperm, oocyte, pre-implantation embryos and trophoblast cells show a reduced or no expression of MHC class I as well as a lack of MHC class II molecules. The lack of human lymphocyte antigen (HLA)-A, -B, and MHC class II expression in trophoblast cells provide mechanisms by which these cells escape maternal immune recognition [6]. Similarly, the loss of MHC class I expression in tumour cells has allowed tumour survival and hindered the rejection by host immune system [7], [8]. Defects in the expression of some components of the antigen processing machinery (APM), such as transporter associated with antigen processing (TAP1/2), low molecular mass protein (LMP2, LMP7) or tapasin (TPN) genes have occurred at the epigenetic, transcriptional and posttranscriptional level [9]. Additionally, a deficiency in some proteins involved in MHC class I antigen processing and peptide generation was reported in mesenchymal stem cells (MSCs) [10].

The non classical MHC class I molecules HLA-E, HLA-F and HLA-G display a more restricted expression pattern and have specialized immune regulatory functions. HLA-E exhibits leader peptides derived from other HLA class I molecules and predominantly inhibits NK (Natural Killer) cell functions. HLA-G is mainly expressed in trophoblast cells and promotes tolerance of the fetus by the maternal T and NK cells. Trophoblast cells express HLA-G and –E which serves to prevent destruction by maternal decidual NK cells [11], [12]. Recently, it had been reported that MSC secrete soluble HLA-G, inhibiting the lysis of target cells by CTLs [13].

Although low MHC class I expression hinders recognition by T and B cells, it may also lead to natural killer cell rejection of the transplanted cells. Stimulatory NK cell receptors such as NKG2D can recognize ligands (MICA, -B, ULBPs 1–5) expressed in embryonic stem cells and lead to their elimination [14], [15]. NKG2D is a potent stimulatory receptor which binds to a family of ligands with structural homology to MHC class I proteins [16]. Human ligands for NKG2D are not expressed in adult healthy tissues but can be induced by cellular stress such as DNA damage, inflammation, heat shock, viral infection or malignant transformation [17], [18].

The constitutive expression of MHC class II molecules is restricted to antigen-presenting cells (APCs). Previous studies showed that MHC class II gene expression is regulated by epigenetic mechanisms. For example, the MHC class II transactivator (CIITA) and the regulatory factor X (RFX) proteins serve as focal points for recruiting histone modifying enzymes to MHC class II promoters. CIITA itself is regulated by DNA methylation and histone modifications [19], [20].

Some soluble factors, such as TGF-β [21] and FasL [3] had been proposed to inhibit immune responses by ESCs. Nevertheless, other reports showed that hESCs were rejected after transplantation into wild-type, fully MHC-mismatched recipients [22]–[24], indicating hESCs immunogenicity. Recently, Yachimovich-Cohen et al [25] had demonstrated a new mechanism to explain how hESCs avoid the allorecognition by the host immune system. They attributed the hESCs inhibitory effect to the L-arginine consumption by hESC arginase I, resulting in downregulation of the TCR CD3-ζ chain and T cell unresponsiveness.

Derivation of induced pluripotent stem cells (iPSCs) from adult somatic cells has raised the possibility of their use for cell replacement therapy and potentially avoiding immunological rejection [26]–[28]. The knowledge of the molecular mechanisms that regulates MHC expression on iPSCs may also have an impact on potential use in transplant therapies.

In the present study, we hypothesized that the low expression of MHC class I and absence of MHC class II in hESCs cells was regulated through epigenetic mechanisms. DNA methylation and histone modification analysis of genes involved in the antigen processing pathway revealed a tight epigenetic control in hESCs. Moreover, we demonstrated that MHC expression in iPSCs behave in a similar way to hESCs.

## Results

### Low levels of MHC-I and absence of MHC-II expression in Shef-1 and NTera2 cell lines

**Figure 1:**
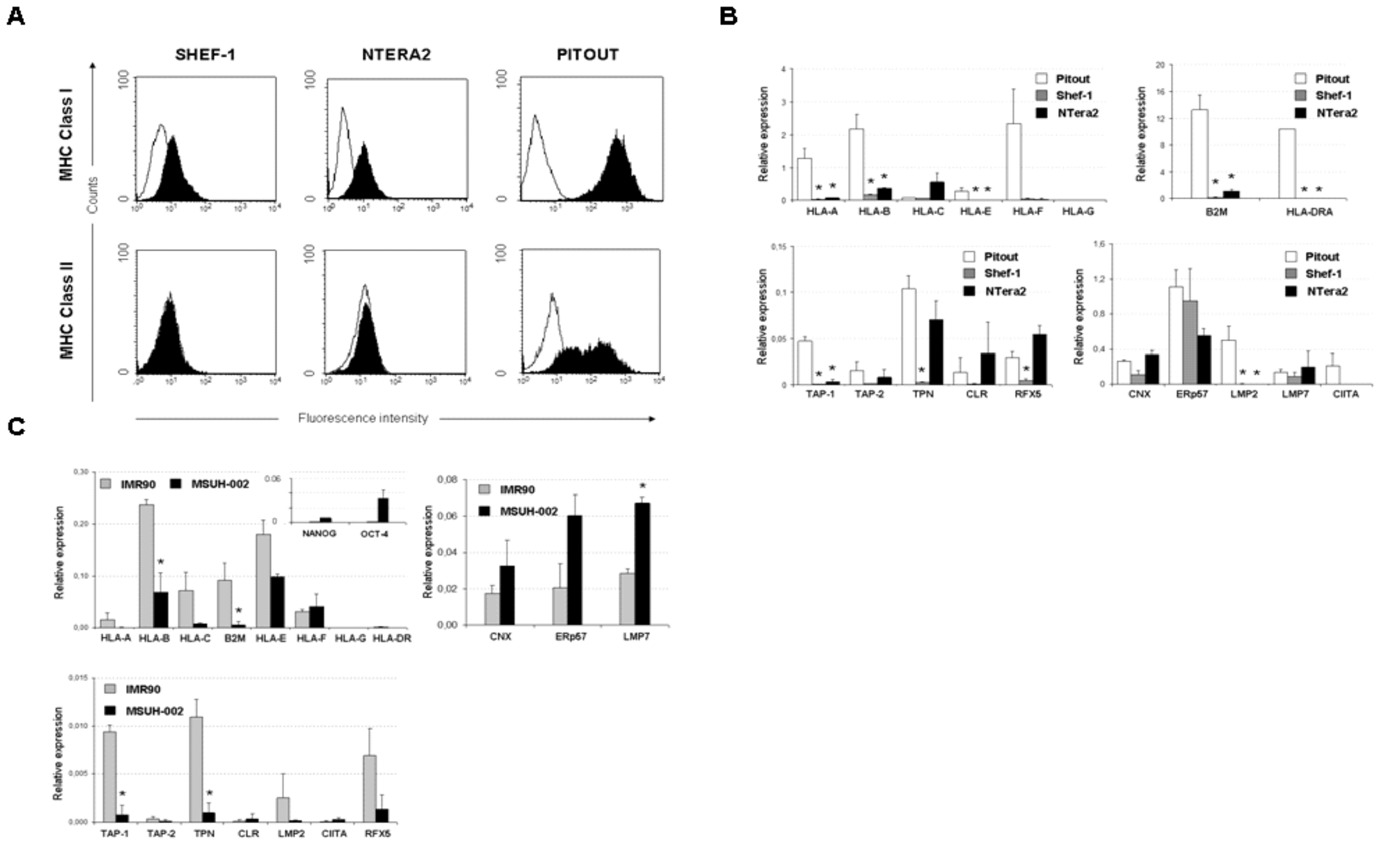
Expression of MHC class I and class II, and the molecules involved in the APM in undifferentiated Shef-1 and NT2 cell lines. **A**) The expression of HLA class I and class II was determined by flow cytometry in undifferentiated Shef-1 and NT2 cell lines. The EBV-transformed B cell line Pitout, which expresses class I and class II, was used as positive control. HLA class I and class II were stained with an anti-pan HLA class I (anti-HLA-ABC mAb)-FITC and HLA-DR-PerCP respectively. Viable cells were gated using 7AAD staining. Thin lines show cells treated with isotype control and black histograms represent positive cells. Similar results were obtained from four independent experiments. **B**) Quantitative-PCR analysis of classical MHC class I molecules (HLA-A,–B and -C), non-classical MHC molecules (HLA-E,-F and –G), antigen processing molecules (TAP-1, TAP-2, TPN, CNX, CLR, ERp57, LMP2, LMP7) and the transcription factors RFX5 and CIITA in undifferentiated Shef-1 and NT2 cells, compared to the control, Pitout cell line. mRNA levels were normalized to GADPH mRNA. **C**) Quantitative-PCR analysis of the MHC genes and APM components in iPSCs and parental IMR90 fibroblast line. The upper right histogram has shown the expression levels of the pluripotent transcription factors Nanog and Oct-4 in fibroblast and iPSCs. mRNA levels were normalized to GADPH mRNA. The results of quantitative PCR are represented as means ± SD from triplicate experiments. * P<0.05.

The expression of MHC class I and class II molecules was analyzed in undifferentiated human embryonic stem cell line Shef-1 and teratocarcioma cell line NT2 by real-time RT-PCR and flow-cytometry. Both cell lines expressed low levels of MHC class I and no expression of MHC class II on the cell surface **(** **Figure 1A** **)**. In agreement with these results, we observed low levels of mRNA transcripts of classical HLA-I molecules (HLA-A and -B) and lack of the non-classical molecules HLA-E, -F and –G in both cell lines **(** **Figure 1B** **)**. Moreover, the mRNA levels of β2-microglobulin (β2m) were nearly undetected in Shef-1 cells but slightly enhanced in NT2 cell line. Additionally, no expression of MHC class II molecules was detected in stem cells.

To determine whether the reduced MHC class I expression might reflect alterations in the antigen processing and presentation in stem cells, we analyzed the expression of APM molecules by real-time PCR. We used the PITOUT cell line, which express MHC class I and class II molecules at high levels, as positive control. Analysis of the APM components revealed that, the transporter molecules TAP-1 and TAP-2 were absent or weakly detected in Shef-1 and NT2 stem cell lines (Fig. 1B). hESC lacked expression of Tapasin (TPN) and Calreticulin (CLR), which are involved in the correct folding of MHC class I molecules, but these molecules were detected at normal levels in the NT2 cell line. Expression of the chaperone molecules, Calnexin (CNX) and ERp57, and the immunoproteasome component LMP7 was commonly detected in both cell lines and exhibited levels comparable to the control cell line. In contrast, lack of the expression of LMP2 was observed. The impaired constitutive expression of MHC class I molecules in Shef-1 and NT2 cells could be due to reduced transcription of some APM components.

### Down-regulation of MHC expression in induced pluripotent stem cells (iPSCs)

Although induced pluripotent stem cells have been proposed as an alternative resource for tissue generation to minimize transplant rejection, their immunological properties are at the moment unknown. We analyzed the MHC expression in the induced pluripotent stem cell line MSUH-002 and its parental human fibroblasts IMR90.

The HLA class I and β2m mRNA levels were lower in iPSCs than in parental fibroblast line **(** **Figure 1C** **)**. HLA-B, -C, -E, and β2m mRNA levels were reduced compared to parental fibroblasts. HLA–F was expressed at similar levels in both cell types whilst HLA-A and HLA-G expression was absent in stem cells.

Regarding the APM genes, TAP-1, TPN, LMP2 and RFX5 mRNA levels plummeted during the reprogramming process to iPSCs. TAP-2, CLR and CIITA were expressed at very low levels in both cell types. The chaperone molecules, CNX and ERp57, and LMP7, had elevated mRNA levels in iPSCs versus fibroblasts. Similar to hESC, no expression of HLA-G and MHC class II molecules was observed. In short, iPSC show reduced MHC class I expression with respect to its parental fibroblast line, suggesting down-regulation of these genes during the cellular reprogramming process.

### Increase of MHC expression during the differentiation process

**Figure 2:**
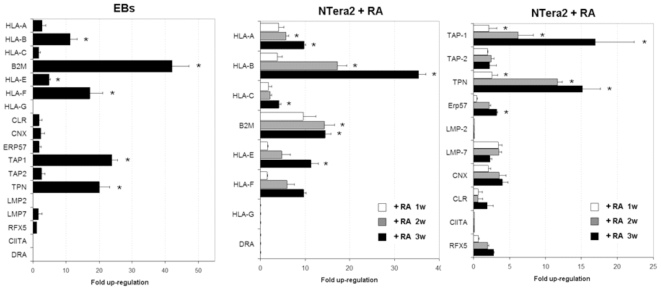
Up-regulation of MHC genes during the differentiation process. Expression levels of the MHC genes and APM components were analyzed by quantitative RT-PCR at different times during the differentiation process to EBs and neuronal precursors. Results are represented as fold up-regulation for each gene in differentiated cells compared to their undifferentiated cells. The results of quantitative PCR are represented as means ± SD from triplicate experiments. * P<0.05. Abbreviation; w: week; RA: Retinoic acid.

To determine the effect of differentiation on MHC expression, Shef-1 cell line was differentiated to embryoid bodies (EBs) and NT2 cells to neuronal progenitors **(Figure S1)**. We compared the expression of MHC and APM molecules on undifferentiated and differentiated cells **(** **Figure 2** **)**. EBs displayed significantly higher expression of HLA-I classical molecule, HLA-B, and the non-classical HLA-E and –F compared to the undifferentiated cells. β2m showed the highest increase of 42 fold as a result of the differentiation process. We proposed that a possible mechanism of the MHC class-I up-regulation could be augmented expression and/or the activation of the APM components. Expression of TAP-1, TAP-2, TPN, ERp57, CNX, CLR and LMP7 genes was induced in differentiated cells, although the level of up-regulation varied. TAP-1 and TPN increased over 23 and 20 times respectively, compared to undifferentiated Shef-1 cells. In contrast, differentiation did not induce HLA-G, LMP-2, HLA-DR, and the transcription factor CIITA mRNA expression. These data inferred that these molecules might be repressed in a stable manner in hESCs or regulated by other mechanisms. Similarly, the NT2 cell line showed the highest up-regulation in HLA-B, β2m, TAP-1 and TPN genes. However, in contrast to hESCs, RFX5 increased slightly during the differentiation process with retinoic acid (RA).

In conclusion, β2m, TAP-1 and TPN increased significantly during differentiation, suggesting that these genes could be responsible for a limited HLA class I expression in undifferentiated human stem cells.

### Expression of NKG2D ligands in human stem cell lines

**Figure 3:**
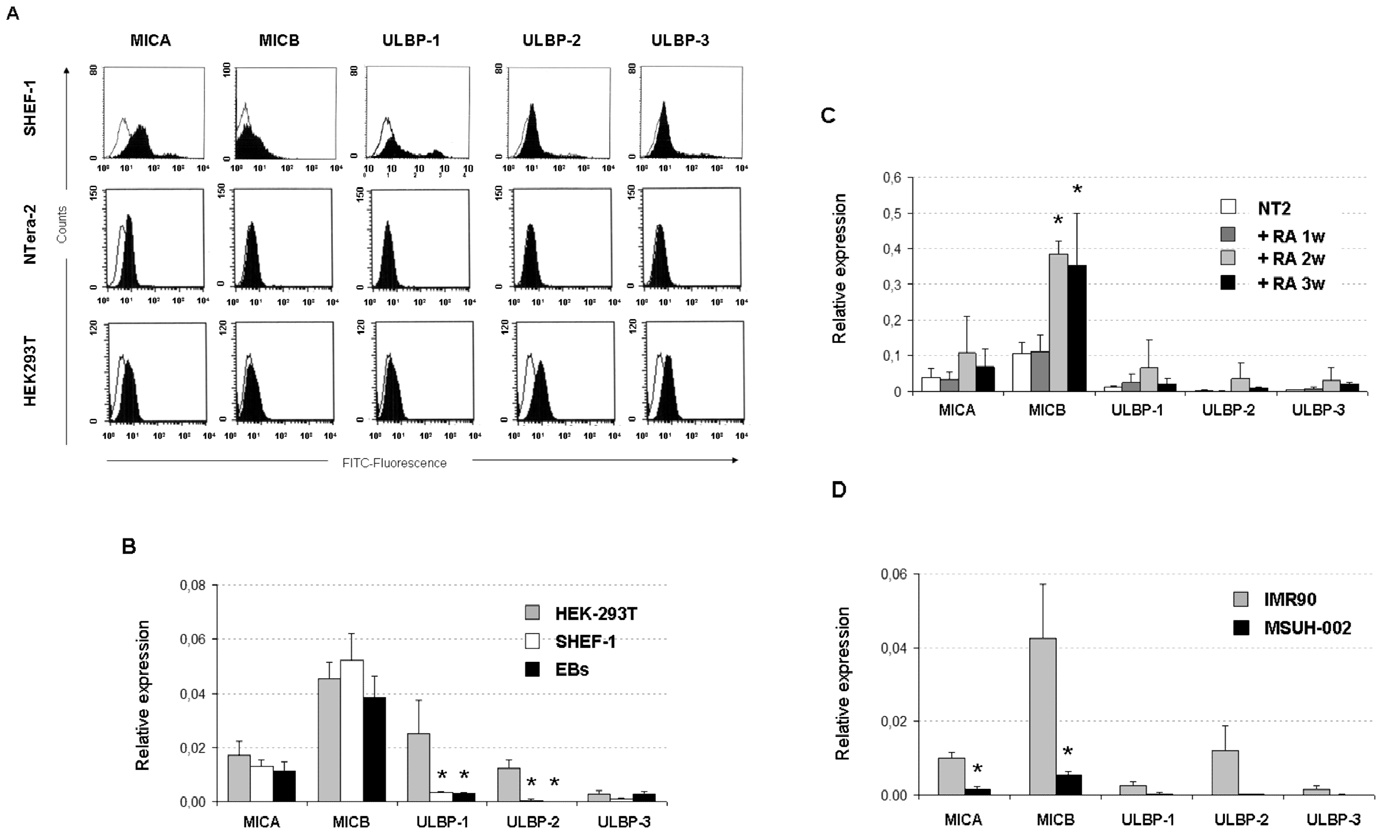
Expression of NKG2D ligands for natural killer cells in undifferentiated and differentiated human stem cells. (**A**) The expression of NKG2D ligands was analyzed in undifferentiated Shef-1 and NT2 cells by flow cytometry using monoclonal antibodies against MICA, MICB, ULBP-1, ULBP-2 and ULBP-3 (1 µg of mAb for sample) followed by FITC-conjugated goat anti-mouse as secondary reagent. Dead cells were excluded by staining with 7AAD. Isotype controls were shown by thin lines and black histograms represented expression of each specific antibody. The HEK-293T cell line was used as positive control. All experiments were performed at least two - three times with similar results. Transcript levels of NKG2D ligands were analyzed by quantitative RT-PCR in undifferentiated human stem cells and during the differentiation process. Undifferentiated Shef-1 (**B**) and NT2 (**C**) cells were differentiated to EBs and neuronal precursors, respectively and the expression for NKG2D ligands was analyzed. The induced pluripotent stem cell line, MSUH-002 (**D**) was compared to parental fibroblast line. The HEK-293T cell line, which expresses mRNA of all the NKG2D ligands, was used as positive control. Histograms represented the relative expression of each gene normalized against the housekeeping gene GADPH. Data are represented as mean ± SD of three independent experiments. * P<0.05. Abbreviation; w: week; RA: Retinoic acid.

Downregulation of MHC class I molecules on cancer cells can lead to their elimination by NK cells if the target cells express ligands for stimulatory NK cell receptors, such as NKG2D ligands (MICA-B, ULBPs 1–5). Analysis of the expression of NKG2D ligands on the cell surface of Shef-1 and NT2 cell lines detected only low expression of MICA proteins **(** **Figure 3A** **)**. Quantitative PCR analyses of NKG2D-L revealed that both cell lines express MICA and MICB mRNA transcripts whilst ULBPs 1–3 were absent or weakly expressed in these stem cell lines **(** **Figure 3B, C** **)**. The human embryonic kidney HEK-293T cell line, which expressed all NKG2D-L mRNA transcripts was used as positive control. These results indicated that expression of NKG2D-L in stem cells could be regulated post-transcriptionally.

We further studied if differentiation was associated with differences in the expression levels of NKG2D ligands. During the differentiation process of hESC to embryoid bodies, most NKG2D-L were maintained in EBs at levels similar to undifferentiated cells, and only MICB expression was slightly reduced **(** **Figure 3B** **)**. Additionally, we differentiated the NT2 teratocarcinoma cell line *in vitro* by the addition of RA to the cultures for 3 weeks and compared the expression levels of NKG2D-L between undifferentiated and differentiated cells **(** **Figure 3C** **)**. Treatment with RA significantly increased MICB mRNA expression in a time dependent manner. However, the increased MICB transcripts were not correlated with high levels of MICB protein on cell surface (data not shown), suggesting additional mechanisms of regulation in this molecule.

Human IMR90 fibroblasts, which were the source for the iPSCs, expressed all NKG2D-L **(** **Figure 3D** **)** but only MICA and MICB expression were detected by real-time RT-PCR in iPSCs, suggesting down-regulation of these ligands during the reprogramming process to pluripotent stem cells.

### Expression of MHC and APM genes is up-regulated by TSA and 5-azaC

To examine the possibility that some MHC class I processing pathway components were epigenetically regulated in hESCs, cells were treated with the epigenetic inhibitors, Trichostatin A (TSA) and 5-azacytidine (5aza-C). IFN-γ, which was reported to induce MHC class I and II expression, was used as control.

**Figure 4:**
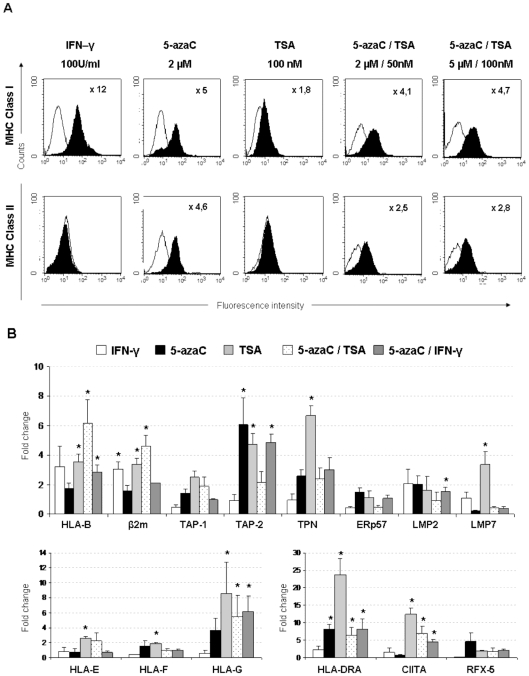
Epigenetic treatments enhanced MHC class I and II expression in undifferentiated embryonic stem cells. **A**) MHC class I and II cell-surface expression in NT2 cells. Cells were treated with IFN-γ (100 U/ml for 24 h), 5aza-C (2 µM for 48 h), TSA (100 nM for 24 h) or a combination of both. After 24 h in culture, cells were stained with specific monoclonal antibodies for MHC class I and II or isotype controls and analyzed by flow cytometry. Isotype controls were shown by thin lines and black histograms represented expression detected by each specific antibody. The proportions of living, dead and apoptotic cells were determined with 7AAD and Annexin V-FITC. Similar results were obtained from three independent experiments. **B**) Quantitative PCR analysis of mRNA levels of MHC class I, class II and APM components in Shef-1 cells treated with epigenetic agents. Cells were cultured with 5aza-C (10 µM), TSA (100 nM) and IFN-γ (100 U/ml) alone, or in combination for 6 hours, and maintained for additional 24 h before RNA extraction. The histograms represented the fold increase of each gene after treatment compared to the basal level of untreated undifferentiated Shef-1 cells. Results were mean ± SD from three independent cultures. * P<0.05.

MHC class I expression was up-regulated in NT2 cells treated with IFN-γ and 5aza-C predominantly **(** **Figure 4A** **)**, but the expression of MHC class II was only increased by exposure to the inhibitor of DNA methyltransferase, 5aza-C. Notably, the effects of TSA and 5aza-C were not synergistic. These results suggested that the absence of MHC class II expression might be due to DNA methylation of these genes or the molecules involved in their regulation.

The effects of epigenetic inhibitors on MHC class I expression were investigated by quantitative real-time RT-PCR in Shef-1 cell line **(** **Figure 4B** **)**. Treatment with 5aza-C significantly increased the expression of TAP-2 gene but only modestly augmented most APM genes. The histone deacetylase inhibitor (HDACi), trichostatin A (TSA), promoted acetylation of histones by inhibiting HDAC and is generally associated with enhanced transcription. Here, we showed that TSA treatment altered MHC class I expression. HLA-B, β2m, TAP-2, TPN and LMP7 expression was significantly enhanced after treatment with 100 nM of TSA. Combined effects of both epigenetic inhibitors were only observed in HLA-B and β2m genes. These results suggested that epigenetic mechanisms may be involved in the regulation of genes required for correct antigen processing and presentation of MHC class I molecules on cell surface of hESCs.

While alone IFN-γ-treatment did not significantly increase the expression of the non-classical MHC class I molecule HLA-G, cells treated with epigenetic inhibitors alone or in combination significantly augmented HLA-G expression, inferring that epigenetic modifications were necessary for the correct expression of HLA-G. Similarly, MHC class II molecules (HLA-DRA) and the essential transcription factor for its expression, CIITA, were significantly induced after treatment with epigenetic modifiers. No relevant changes in the expression of RFX5 were observed.

Previous studies in tumour cells have shown that the expression of IFN-γ-inducible genes can be enhanced by treatment with epigenetic inhibitors. In our study, we observed that the combined effects of 5aza-C and IFN-γ slightly enhanced the expression of HLA-B and HLA-G compared to cells treated with 5aza-C alone **(** **Figure 4B** **)**. The combination of 5aza-C plus IFN-γ on the CIITA gene exerted a greater than additive effect, suggesting that demethylation of the CIITA promoter may be necessary for the further induction by IFN-γ.

### Methylation of HLA-G, MHC-II and CIITA in hESCs

**Figure 5:**
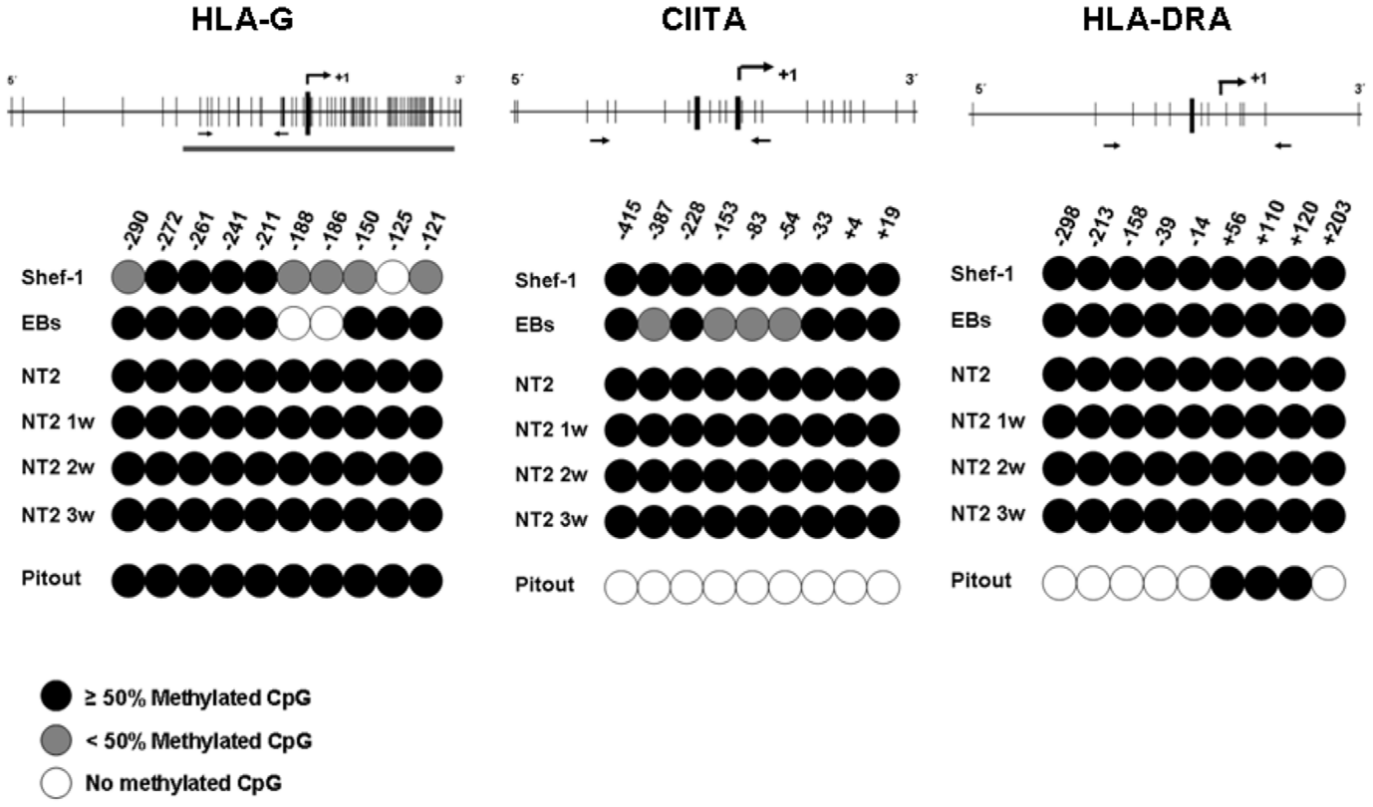
Methylation profile of HLA-G, CIITA and HLA-DRA promoter regions in undifferentiated and differentiated cells by bisulfite sequencing analysis. The upper panels displayed a schematic map of each promoter region studied. The position of each CpG dinucleotide was depicted with thin vertical lines. Transcriptional start site is indicated by a thick vertical line and the +1 position indicated the translation start site. Arrows indicated the amplified region by RT-PCR for bisulfite sequencing analysis. In HLA-G, the horizontal gray line marked the CpG island. Ten clones were sequenced for each sample and each circle represented the average methylation for each CpG dinucleotide (open circle: 100% unmethylated, black circle: ≥50% CpG methylated, gray circle: <50% CpG methylated). The numbers denoted the position of each CpG site studied. Pitout cell line, which express MHC class I and II was used as control.

Methylation is one of the major epigenetic modifications that repress transcription *in vivo*. The methylation state of the genes implicated in the MHC expression (HLA class I and II molecules, and APM components) was assessed with bisulfite modification of isolated genomic DNA from undifferentiated stem cells and its derivative cells. Most genes studied were not methylated in the promoter region, indicating that the transcription of these genes in hESCs was not regulated by DNA methylation. In contrast, HLA-G, HLA-DR and CIITA genes were fully methylated. Profiles of CpG methylation in the regulatory regions of these genes were represented graphically in **Figure 5**. HLA-DR loci were completely methylated at all 9 CpG sites analyzed in Shef-1 and NT2 cells and this methylation status was maintained through the differentiation process. In contrast, the control cell line Pitout expressed MHC class II and displayed no methylation in the promoter region. The transcription factor CIITA, which regulated the expression of MHC class II molecules, was completely methylated in Shef-1, NT2 cells, and neuronal progenitors, although some CpG sites exhibited partial methylation in EBs **(** **Figure 5** **)**. HLA-G gene contained a CpG island around its transcriptional start site. This region was partially methylated in Shef-1 and EBs, and only 4 CpG sites were fully methylated (region −211 to −272). NT2 and derivative cells exhibited full methylation in all HLA-G CpG sites.

Results of bisulfite sequencing were confirmed by methylation arrays **(Figure S2, Table S1)**. The MHC class II genes (HLA-DP, -DQ and –DR) and their transcription factor CIITA were hypermethylated in hES and iPS cells (array signal ≥0.7, red) whereas MHC class I and APM components were demethylated in hES and iPS cells (array signal <0.3, green). Some divergences respects to bisulfite sequencing were observed in HLA-G and TAP-1, probably due to the different regions analyzed by both methods. The specific probe for HLA-G gene overlapped a region (−497 bp) far from the transcription start, and not include CpG sites.

As we show by methylation arrays **(Figure S2)**, profiles of methylation were very similar between induced pluripotency and human embryonic stem cells. Thus, the absence of MHC class II and HLA-G expression in hESC and iPSC may be due to DNA methylation of these genes in combination with the transcription factor, CIITA.

### H3-K4 and H3-K9 methylation profiles in hES and iPS cells

**Figure 6:**
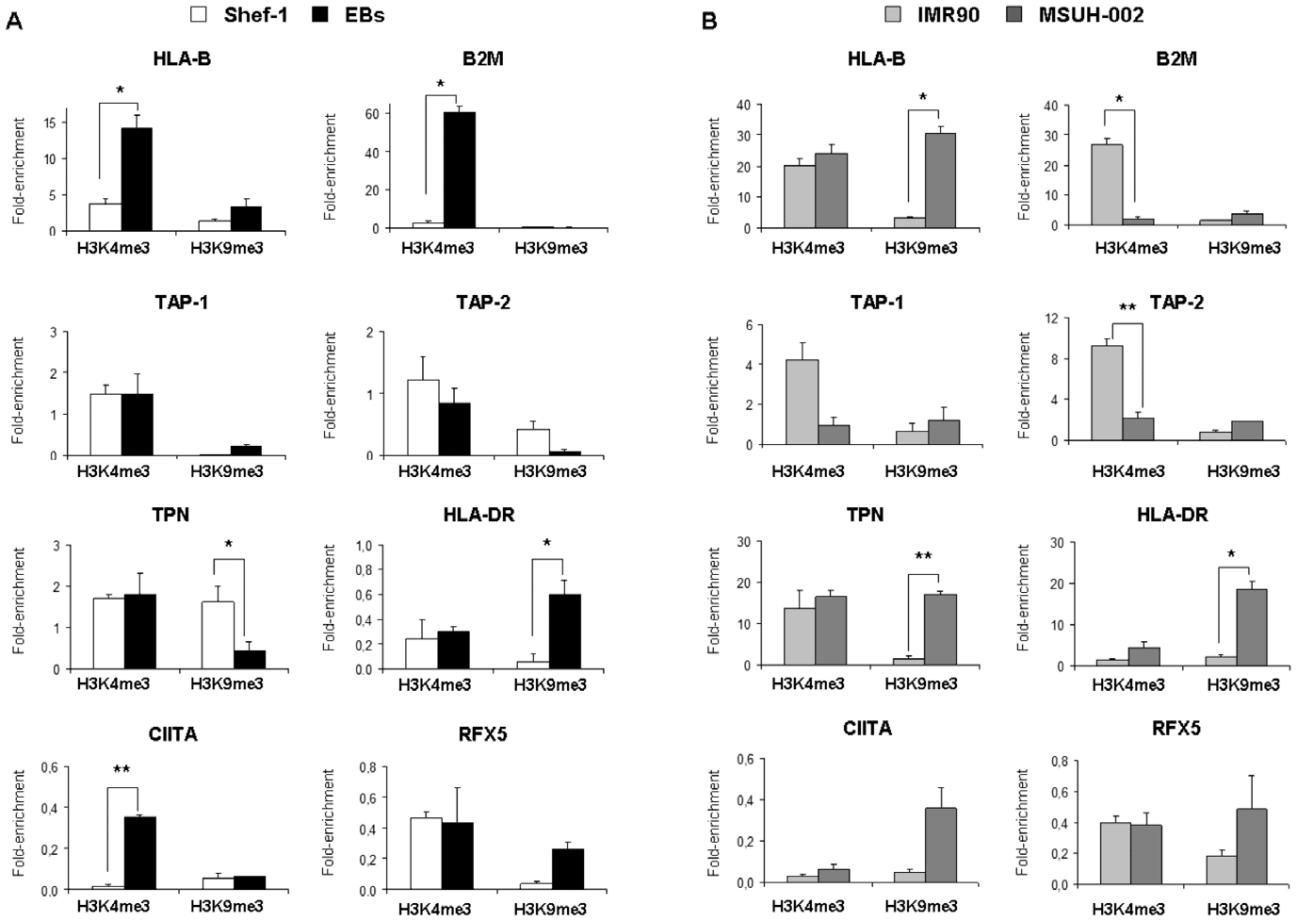
Methylation of histones H3-K4 and H3-K9 regulated MHC expression in hES and iPS cells. Levels of the trimethylation H3-K4 (active mark, H3K4me3) and H3-K9 (repressive mark, H3K9me3) were determined at selected regions of MHC genes and APM components in: **A**) undifferentiated Shef-1 cells (white columns), or embryoid bodies (EBs) (black columns) formed after 10–15 d differentiation in culture and **B**) IMR90 human fibroblast (light gray columns) and MSUH-002 cells (dark gray columns). Cells were subjected to ChIP assay using antibodies against H3K4me3 and H3K9me3, and analyzed by quantitative PCR with specific primers for each gene. Normal rabbit IgG was used as negative control for the specificity of the immunoprecipitation (IP). As a positive control, aliquots of chromatin fragments obtained before IP were also subjected to Q-PCR analysis (Input). Immunoprecipitated DNA associated with a given histone modification was normalized to a 100-fold dilution of input chromatin. Data are expressed as fold enrichment of each modification compared to negative control antibody (normal rabbit IgG), and represented mean ± SD of two representative experiments with similar results (*P<0.05: **P<0.01).

Posttranslational modifications of the amino-terminal tails of core histones also contribute to regulation of gene expression. To determine whether histone modifications regulate MHC transcription during the differentiation process, we performed ChIP assays using antibodies against the marks H3K4me3 (active) and H3K9me3 (repressive). H3K4 trimethylation at the HLA-B promoter was higher in EBs than in undifferentiated Shef-1 cells **(** **Figure 6A** **)**, whereas H3K9me3 was similar in both cell types. Similarly, the level of H3K4 trimethylation in β2m gene was 60 fold higher in EBs than in Shef-1 cells, and corresponded to the high increase of β2m mRNA in EBs. These results indicated that the histone modification H3K4me3 facilitated chromatin relaxation in HLA-B and β2m genes during the differentiation process and allowed its expression. CIITA gene also showed high levels of H3K4me3 in EBs, suggesting a possible increased mRNA transcript level in these cells. In respect to the APM genes, H3K4me3 of TAP-1, TAP-2 and TPN genes was similar in undifferentiated and differentiated cells. However, the repressive mark was significantly higher on TPN in Shef-1 cells than in EBs, and corresponded to undetectable TPN levels in undifferentiated cells and higher TPN levels in differentiated EBs. This modification may hinder their transcription by maintaining a compact state of chromatin in undifferentiated cells,. The limited HLA-DR and RFX5 expression during the differentiation process may reflect the high H3K9me3 in the promoter region in EBs. We did not find any mark in the promoter region of the non-classical MHC class I genes (HLA-E, -F and –G) (data not shown), suggesting that other histone modifications or mechanisms were involved in their regulation.

Histone modifications in fibroblasts on HLA-B, β2m, and some APM genes exhibited activation marks congruent with their expression **(** **Figure 6B** **)**. In contrast to hESCs, iPSCs exhibited the repressive mark H3K9me3 at the HLA-B promoter, which correlated with the reduced expression of this gene. Histone modifications in TPN gene were similar between iPSCs and hESCs, and high levels of both marks were observed, consistent with its repression in undifferentiated stem cells. However, H3K9me3 was higher in the promoter region of HLA-DR, CIITA and RFX5 in iPSCs than in hESCs.

Thus, HLA-B, β2m, HLA-DR, TPN and CIITA might be regulated in hESCs by histone modifications, such as H3K4me3 and H3K9me3 marks. Additionally, we verified that HLA-B, TPN, HLA-DR, CIITA and RFX5 acquired repressive marks which suggested chromatin was remodelled during the reprogramming process from human somatic cells to induced pluripotency stem cells.

## Discussion

Overcoming the immunological barriers to the stem cell transplantation is one of the most important clinical challenges, and will change the future of regenerative medicine and cellular therapy. Therefore, it is critical to understand the immunogenicity of hESCs, and the necessary modifications to induce acceptance of these cells by the patient's immune system. Several approaches had been proposed to overcome graft rejection, such as development of hESCs banks, nuclear transfer, or the creation of a universal stem cell line [29]. The iPSCs technology potentially could overcome two important problems associated with human hESCs: ethical problems based on the use of human embryos and immune rejection after transplantation [30], although little is known so far about the immunogenicity of these new pluripotent stem cells.

In this report, we demonstrated that hESCs expressed low levels of classical HLA-class I and absence of HLA-class II molecules on the cell surface. Analogous expression levels were observed in human iPSCs, suggesting down-regulation of these molecules during the cellular reprogramming process from human adult fibroblast. Furthermore, pluripotent stem cells (Shef-1, NT2 and MSUH-002 cell lines) show absent or reduced expression of β2-microglobulin light chain, which could limit the expression of the MHC class I trimeric molecule on the cell surface. Similarly with tumour and trophoblast cells, the absence of MHC class I expression represents a mechanism of immune evasion in these cells.

We hypothesized that low levels of MHC class I molecules could be due to defects in the APM components in stem cells. Expression of TAP-1, TAP-2 and TPN molecules, which are implicated in the transport and load of peptides onto MHC class I molecules, was weakly detected in embryonic and induced pluripotent stem cells. Unexpectedly, the NT2 teratocarcinoma cell line shows a low expression of TAP-1 and TAP-2, but express tapasin at normal levels. The lack of the TAP-1/TAP-2 complex in hESCs implied that the pool of endogenous peptides can not bind to MHC class I dimers, and inhibited the MHC class I heavy and light chain complexes from leaving the endoplasmic reticulum. TAP-1 knockout mice show significantly reduced levels of MHC class I surface expression [31]. In addition, the chaperones ERp57, CNX and the immunoproteasome component LMP7 were commonly expressed at high levels by all stem cell lines, indicating that the folding of MHC molecules and generation of antigenic peptides in these cells were not damaged. Taken together, our results confirmed that pluripotent stem cells were partially defective in their ability to process and present MHC class I molecules on the cell surface. These results are in line with a previous report which show that the HS293 hESC line lack the expression of some APM components [32].

During the differentiation process to EBs, most HLA class I molecules and APM components except HLA-G and LMP2 increased significantly compared to undifferentiated Shef-1 cells. Additionally, β2m protein, TAP-1 and TPN increased strongly upon differentiation, suggesting that the up-regulation of MHC class I in differentiated cells might be a consequence of the increased expression of these genes during the differentiation process. However, no changes in the expression of MHC class II and CIITA were observed. Thus, increased expression of MHC class I in differentiated stem cells may lead to the recognition and elimination by T cells, representing the most important barrier for transplantation of pluripotent stem-cell-derived allografts.

That is the first time that NKG2D ligands were analyzed in human stem cells. Expression of MICA and MICB genes at RNA level was found in Shef-1 and NT2 cell lines and maintained during the differentiation to EBs and neuronal progenitors, respectively. ULBP proteins were weakly expressed and only slightly increased during the differentiation with RA. iPSCs showed a similar pattern of NKG2D-L expression, although it was not possible in this study to determine the protein level on the cell surface. Human stem cells with MICA and MICB expression may be recognized and lyzed by NK cells even in the presence of inhibitory MHC class I molecules. Previous studies have reported that mouse embryonic stem cells are resistant to lysis by NK cells [33] while Dressel et al [34] showed that susceptibility to lysis by NK cells mediated by NKG2D ligands is a common feature of pluripotent murine stem cells. Differences between these studies may be explained by differences in the activation status of the NK cells. Recently, Di Tomaso et al [35] have demonstrated that cancer stem cells (CSCs) isolated from human tumours were negative for NKG2D ligands. Treatment of CSCs with the demethylating agent, 5aza-C, restored the expression of these molecules, and led to their recognition and lysis by NK cells.

Nevertheless, in our study, no cell surface expression of NKG2D-L was detected on Shef-1 and NT2 cell line except low levels of MICA. These results suggest that NKG2D ligands might be regulated at the post-transcriptional level in stem cells. Several microRNAs have suppressed the expression of MICA and MICB proteins below a certain threshold and facilitated an acute up-regulation during cellular stress [36], [37]. Furthermore, MICB has a shorter half-life at the plasma membrane than MHC molecules [38]. MICB expression depends on its recycling in trans-Golgi network and late endosome-related compartments as well as shedding into the extracellular medium.

Expression of NKG2D-L at RNA level but no protein expression was detected on the cell surface of trophoblast cells [39], [40], showing a permanent shedding of these ligands from trophoblast cells mediating the maternal-fetal tolerance. Additional studies have confirmed that exosomes bearing NKG2D-L are released by human placenta and tumour cells and induce down-regulation of the NKG2D receptor on NK and CD8+ T cells which inhibited their cytolytic capability [41], [42]. Our data suggested that human stem cells may use similar mechanisms to evade the recognition by NK cells and contribute to the immune-privileged properties of these cells. Further studies are warranted to verify the role and regulation of NKG2D ligands in hESC.

Epigenetic modifications, such as hypermethylation of promoter regions or histone modifications may regulate the expression of MHC class I and II, and APM molecules in human pluripotent stem cells. To confirm this, we cultured hESCs *in vitro* with 5aza-C and TSA, showing that epigenetic mechanisms alter the expression of all APM components involved in the antigen processing and presentation. We observed that expression of HLA-DR and CIITA was only induced by the treatment with epigenetic agents but not during the differentiation. These data suggested that direct or indirect epigenetic modifications were required to restore their expression. Absence of MHC class II molecules in hESCs was associated with hypermethylation in the CIITA promoter region, in addition to methylation in the promoter region of the HLA-DRA gene. Moreover, methylation of CIITA may be at least partially responsible for the low levels of MHC class I expression, as transfection of trophoblast cells with CIITA has restored MHC class I expression [43]. Additionally, HLA-G promoter was fully methylated in 4 CpG sites in hESCs and EBs, so this region could be directly implicated in the regulation of HLA-G. In agreement with our results, it has been recently reported that the 4 CpG sites in the HLA-G promoter region contained a hypoxia response element (HRE) that remained completely methylated in ovarian cancer cell lines [44]. Thus, methylation of HLA-DR and HLA-G promoters in human stem cells contributed to the restricted expression of these genes in somatic cells.

Modifications of histones proteins are responsible for a “histone code” that epigenetically regulates chromatin and gene expression [45]. We have analyzed some of these histone marks to determine whether they are involved in the regulation of APM components. H3K4me3, a histone mark that facilitates the binding of transcription factors, was present at high levels in HLA-B and β2m gene promoters in EBs in comparison to undifferentiated Shef-1 cells. Although no H3K9me3 was observed in undifferentiated cells, other repressive marks not analyzed here could be involved in the repression of these genes. Absence of TPN expression in undifferentiated hESCs might be due to the presence of the repression mark H3K9me3, which is present at lower levels in differentiated cells. Tapasin is involved in the stabilization of TAP-1/2 complex and peptide loading in MHC class-I molecules, and could act as a limiting factor in the expression of these molecules in hES cells. Furthermore, this repressive mark was acquired when human IMR90 fibroblasts were reprogrammed into iPSCs, indicating that epigenetic changes in MHC genes occur during the cellular reprogramming process.

Absence of MHC class II induction in EBs can be associated with H3K9me3 in HLA-DR, although we demonstrated above that other mechanisms such as DNA methylation can also participate in their repression. The active mark, H3K4me3, was present in CIITA gene in EBs and facilitated the transcription process during differentiation. However, we now know that the CIITA promoter was methylated in human stem cells. Additionally, the HLA-DR gene and the transcription factors CIITA and RFX5 exhibited high levels of H3K9me3 in iPSCs. These data indicated that these genes could be silenced during the reprogramming process by repressive histone modifications in addition to DNA methylation. In fact, it has been described previously that trimethylation of H3K9 is associated with DNA methylation [46]. This is the first time that histone modifications (H3K4me3 and H3K9me3) were described in APM components and implicated in the low expression of MHC class-I or absence of MHC class-II molecules in hESCs. Moreover, the analogous distribution of histone modifications between hESC and iPSCs suggest that changes detected in regulatory regions of MHC genes are indicative of remodelling chromatin on APM promoters to acquire an epigenetic state characteristic of pluripotent cells. Likewise, murine iPSCs were highly similar in their epigenetic state to ES cells, showing that transcription factor-induced reprogramming leads to the global reversion of the somatic epigenome into an ES-like state [47]. Further studies with other histone modifications will be necessary to elucidate the complete epigenetic regulation of these genes in pluripotent stem cells.

In short, the lack or down-regulation of MHC molecules in hESCs is due to absence of some APM mRNA transcription. The differentiation process augments mRNA transcription of APM components which yield an increase of MHC class I on cell surface. This process is regulated by modifications in chromatin remodelling, mainly H3K4me3 in HLA-B, and β2m, as well as H3K9me3 in TPN gene, respectively. Moreover, DNA methylation profile matched the absence of MHC class-II and the tolerogenic molecule HLA-G expression in undifferentiated and differentiated hES cells. We conclude that epigenetic modifications regulate MHC class I and class II expression in hESCs and iPSCs, similar to trophoblast and tumour cells. Reduced MHC class I and class II expression in hESCs and iPSCs can limit their recognition by the immune response against these cells. The knowledge of these mechanisms will further the development of strategies to induce tolerance and improve acceptance of stem cell allografts.

## Materials and Methods

### Cell lines

The lymphoblastic cell line PITOUT was grown in RPMI medium supplemented with 10% heat-inactivated fetal bovine serum (FBS), 2 mM L-glutamine, 100 U/ml penicillin and 100 µg/ml streptomycin. HEK-293T (human embryonic kidney 293T cells) cell lines were maintained in supplemented DMEM medium.

### Embryonic stem cells culture, differentiation and treatment

The hES cell line, Shef-1 [48] was maintained in hESCs medium, Knock-Out DMEM medium supplemented with 20% Knock-Out Serum Replacement on inactivated mouse embryonic fibroblast (MEF) as previously described [49], [50]. For differentiation to embryoid bodies (EBs), hES Shef-1 colonies were cultured in suspension in hESCs medium without bFGF-2 for 10–15 days. The human embryonal carcinoma cell line NTera2 clone D1 (NT2) was cultured in DMEM medium +10% FBS and differentiated to neuronal progenitors with 10 µM all-trans-retinoic acid (Sigma-Aldrich) for 3–4 weeks as previously described [51]. Culture and characterization of Shef-1 and NT2 cell lines was detailed in **Methods S1** and **Figure S1**.

Shef-1 and NT2 cells were cultured in ES medium supplemented with 5-Aza-Cytidine (5azaC) and/or Trichostatin A (TSA) (Sigma-Aldrich) at indicated concentrations. Subsequently, cells were cultured in fresh medium for 24 h before RNA extraction. As control, Interferon-γ was used at 100 U/ml.

### Induced Pluripotent stem cells

DNA and RNA from the human induced Pluripotent Stem Cell (iPSC) Line, MSUH-002, were kindly gifted by Professor J. Cibelly at the University of Michigan University. These stem cells were produced from IMR90 cells as previously described [28]. Briefly, viral vectors were packed in HEK-293T cells. The resulting viral particles were concentrated by ultracentrifugation and the viral transductions were performed in hESCs media. iPSCs colonies were manually detached and expanded as individual clones in the same media.

### Immunofluorescence

hES cells were fixed with 4% paraformaldehyde and stained with the primary antibodies SSEA-3 (1∶40), SSEA-1 (1∶10) and TRA1-60 (1∶100) (kindly gifted by Professor Moore, Sheffield, UK) overnight at 4°C followed by incubation with FITC-conjugated second antibodies for 1 h.

### Reverse transcription-PCR and real-time RT-PCR

Total RNA was isolated using the RNeasy kit (Qiagen, Valencia, CA) according to the manufacturer's instructions and reverse transcribed using the iScriptTM cDNA Synthesis kit (BioRad, Hercules, CA). Real-time PCR assay was performed in 20 µl of SYBR Green Super Mix (Bio-Rad) using a MyiQ Single-Color Real-Time PCR Detection System (Bio-Rad). DNA was denatured at 95°C for 30 seconds, annealing at 60°C for 1 min, elongation at 72°C for 1 min and extension at 72°C for 5 min. Primers used are reported in **Table S2**. Fold changes in transcript levels were calculated using threshold cycle (Ct) values standardized to GAPDH, which was used as the endogenous control. All samples were run in triplicate and at least two independent experiments were carried out.

### Flow cytometry analysis

hES colonies were harvested with 0.1% collagenase IV (Invitrogen, CA), dissociated into single cells and stained with the monoclonal antibodies FITC-conjugated anti-pan HLA class I (anti-HLA-ABC mAb) and PerCP-conjugated HLA-DR (BD Biosciences). Analysis was carried out in a FACScan Cytometer (Becton Dickinson). Antibodies against NKG2D ligands (MICA, MICB, ULBP 1–3) (R&D Systems) were used at 1 µg/ml and FITC goat anti-mouse (eBioscience) was used as secondary antibody. The proportions of living, dead and apoptotic cells were determined with 7AAD and the Annexin V-FITC apoptosis detection kit (Immunostep Inc; Spain).

### DNA methylation array

Methylation was assessed at 1,505 CpG sites using Illumina Goldengate Methylation Arrays© and analyzed as we previously described [**48**]. Details are reported in **Methods S1**.

### Bisulfite modification

DNA methylation was determined by PCR analysis after bisulfite modification of the DNA. Genomic DNA was purified using the DNeasy Blood and Tissue Kit (Qiagen, Valencia, CA) according to manufacturer's recommendations. DNA bisulfite modification was performed using the EZ-DNA Methylation kit (Zymo Research, CA) and the DNA was amplified using specific primers designed with the Methyl Primer Express Software® (Applied Biosystems). Primers were shown in **Table S3**. PCR products were cloned into pGEM-T Easy Vector System II (Promega, Madison, WI) and DNA plasmids purified with the QIAprep Spin Miniprep Kit (Qiagen). In each case, ten independent clones were automatically sequenced to determine their degree of methylation.

### Chromatin immunoprecipitation (ChIP) assay

Chromatin immunoprecipitation assays using 0.5-1×10^6^ cells per sample were performed as previously described [52] with the anti-trymethylated H3K4 and H3K9 (Upstate Biotechnologies.Inc) antibodies. Normal IgG was used as negative control. In brief, fixed cells with 1% formaldehyde were lysed in SDS-lysis buffer (1%SDS, 10 mM EDTA, 50 mM Tris-HCl pH 8.1) and sonicated. The shared chromatin were diluted into ChIP dilution buffer (0,01% SDS, 1,1% Triton X100, 1,2 mM EDTA, 16,7 mM Tris-HCl pH 8,1, 167 mM NaCl) and incubated with the antibodies overnight at 4°C. Antibody-chromatin complexes were precipitated with Salmon Sperm DNA/Protein A-Agarose beads (Upstate Biotechnologies), washed and eluted from the beads using elution buffer (1% SDS, 0.1 M Na HCO_3_). After cross-link reversal and proteinase K treatment, DNA was extracted with phenol-chloroform and ethanol precipitated. Immunoprecipitated DNA was analyzed in triplicate by real-time PCR from 1 µl of eluted DNA. Primers for each promoter were listed in **Table S4**. Aliquots of chromatin obtained before immunoprecipitation were analyzed as input control. Results are presented as fold enrichment of precipitated DNA associated with a given histone modification, relative to a 1/100 dilution of input chromatin.

### Statistics

Statistics were calculated using SPSS Student version 14.0 software. Student's t test was used for statistical analysis. A P value of <0.05 was considered significant.

## Supporting Information

Methods S1 (0.03 MB DOC)

Figure S1 Culture and characterization of human embryonic stem cell line Shef-1 and the human carcinoma cell line NTera2 and differentiated cells. A) Culture of Shef-1 hES cell line and differentiation to embryoid bodyes (EBs). Shef-1 cells were grown in mouse embryonal fibroblast (MEFs) in gelatine-coated dishes in complete hESC medium with bFGF-2 (a). To differentiate to EBs (c), colonies were detached from MEFs and culture in suspension without bFGF-2 for 15 days. Panels b and d displayed hESC and EBs from Shef-1 cells respectively, at high magnification. B) Immunofluorescence analysis of specific cell-surface antigens, SSEA-1, SSEA-3 and TRA1-60 in Shef-1 hESC. Undifferentiated cells were strongly positive for SSEA-3 and TRA1-60 and only differentiated cells were stained by SSEA-1 antibodies. C) Induction of neuronal morphology in NT2 cell line. Undifferentiated cells (a) were treated with retinoic acid (RA) for 3–4 weeks. Samples were taken 1 week (b) and 3 weeks (c,d) and neuronal progenitors were observed. D) RT-PCR analysis of in vitro differentiated hESCs to EBs for detection of expressed genes of the three embryonic germ layers, enolase (mesoderm), amylase (endoderm) and neurofilament (ectoderm) and pluripitency genes (Nanog and Oct-4). E) RT-PCR analysis during the differentiation process of NT2 cell line to neuronal progenitors, Differentiated cells express well-characterized neuronal markers such as Tau or NeuroD whilst Nestin, a typical neuroectodermal marker, was downregulated by RA treatment. The transcription factors Nanog and Oct-4 were lost after 1 week in culture showing a right process of neural differentiation.
(5.40 MB TIF)

Figure S2 Methylation profiles of MHC genes and APM components. Methylation profiles of HLA class I, class II, antigen processing machinery (APM) genes and transcription factors (TF) involved in MHC regulation in hESCs, NT2 cell line, iPSCs and IMR90 fibroblast were obtained by Illumina arrays. Cluster analysis was based on correlation of methylation profiles of MHC and PM genes. The methylation levels vary from fully methylated (red) to fully unmethylated (green) sequences.
(0.92 MB TIF)

Table S1 Classification of MHC genes according to their promoter methylation status in hESCs Shef-1, NT2 cell line, iPSC cell line and human fibroblast IMR90. The classification criteria are described in the Methods S1.
(0.07 MB XLS)

Table S2 Primers used for real-time RT-PCR.
(0.06 MB DOC)

Table S3 Primers used for bisulfite sequencing.
(0.04 MB DOC)

Table S4 Primers used for Chromatin Immunoprecipitation (ChIP) assay.
(0.04 MB DOC)
