# Supplementary material for: Epigenetic Mechanisms Regulate MHC and Antigen Processing Molecules in Human Embryonic and Induced Pluripotent Stem Cells
Source: PLoS One. 2010 Apr 16;5(4):e10192. doi: 10.1371/journal.pone.0010192 (PMC2855718; doi:10.1371/journal.pone.0010192)
Supplement: Methods S1 — (0.03 MB DOC) [file pone.0010192.s001.doc]

**METHODS S1**

***Embryonic stem cells culture and differentiation***

The hES cell line, Shef-1 was cultured on mouse embryonic fibroblast (MEF) feeders layers in hESC medium: Knock-Out DMEM medium was supplemented with 20% Knock-Out Serum Replacement, 0,1mM nonessential amino acids, 1mM sodium pyruvate, 0,1mM 2-mercaptoethanol and 4 ng/ml of human recombinant basic fibroblast growth factor (bFGF-2) (Invitrogen). The feeder cells were mitotically inactivated with 10 µg/ml mitomycin C (Sigma, USA). Small colonies were transferred to mitotically MEF feeders layers in T-25 flask and culture continued. To maintain undifferentiated colonies, the media was replacement daily and the cells were split (1:2) and transferred onto new feeders layers every 5 days with 200 U/ml of collagenase (Invitrogen). *In vitro* differentiation of hES cells was carried out by the following procedure. For differentiation to embryoid bodies (EBs), hES Shef-1 colonies were removed from feeder’s cells, digested with 1 mg/ml collagenase and scrubbed into small clumps. The clumps of hESC were transferred to low cell-binding dishes (Nunc) and cultured in suspension in hESC mediun without bFGF-2 for 10-15 days. The medium was replaced every two days, until completely differentiation. After 2 weeks of culture, total RNA was isolated and analyzed by real-time RT-PCR experiments.

The human carcinoma cell line NTera-2 clone D1 (NT2) is similar to embryonic stem cells and has the capacity to differentiate into post-mitotic neurons of the central nervous system upon exposure to retinoic acid (RA). To generate neuronal derivatives or neuronal progenitors, cells were seeded in complete DMEM medium + 10% FCS at 2 x 10^6^ cells per 150mm sterile culture dish. Culture was maintained for 24 h before adding 10 µM all-trans-retinoic acid (Sigma-Aldrich). Cells were cultured for 3-4 weeks in retinoic acid, replacing the medium every 2-3 days. After first week, cells were plated onto poly-L-lysine (10 µg/ml; Sigma-Aldrich)-coated plates in complete medium DMEM containing the mitotic inhibitors fluorodeoxyuridine (10 µg/ml), cytosine arabinoside (1 µM) and uridine (10 µg/ml) (Sigma-Aldrich). Analysis by real-time RT-PCR of neuronal genes (Tau, NeuroD and Nestin) was used to confirm the induction of a neuronal phenotype.

**DNA methylation array**

Methylation was assessed at 1,505 CpG sites using Illumina Goldengate Methylation Arrays©, as described in Bibikova et al. **(1).** The amount of bisulfite-modified target DNA that hybridizes to each spot of the Illumina chip was quantified and standardized to a maximum of 1.0 (the likelihood of hypermethylation of this gene promoter is almost 100%) and a minimum of 0.0 (the likelihood of promoter hypermethylation is almost 0%). In this work, all sequences with at least 70% likelihood of being hypermethylated (hybridization signal ≥ 0.7) were considered hypermethylated for each specific sample, whereas sequences whose equivalent signal was below 30% (hybridization signal < 0.3) were considered unhypermethylated **(Supplementary Table 4).** Cluster analysis of hESCs, EBs, iPSCs and IMR90 fibroblast were based on correlation of methylation profiles of MHC and APM genes **(Supplementary Figure 2).**

**1.** **Bibikova M** et al.(2006) Human embryonic stem cells have a unique epigenetic signature. Genome Res.16:1075-83.
