# Supplementary figures and images for: Epigenetic Mechanisms Regulate MHC and Antigen Processing Molecules in Human Embryonic and Induced Pluripotent Stem Cells
Source: PLoS One. 2010 Apr 16;5(4):e10192. doi: 10.1371/journal.pone.0010192 (PMC2855718; doi:10.1371/journal.pone.0010192)

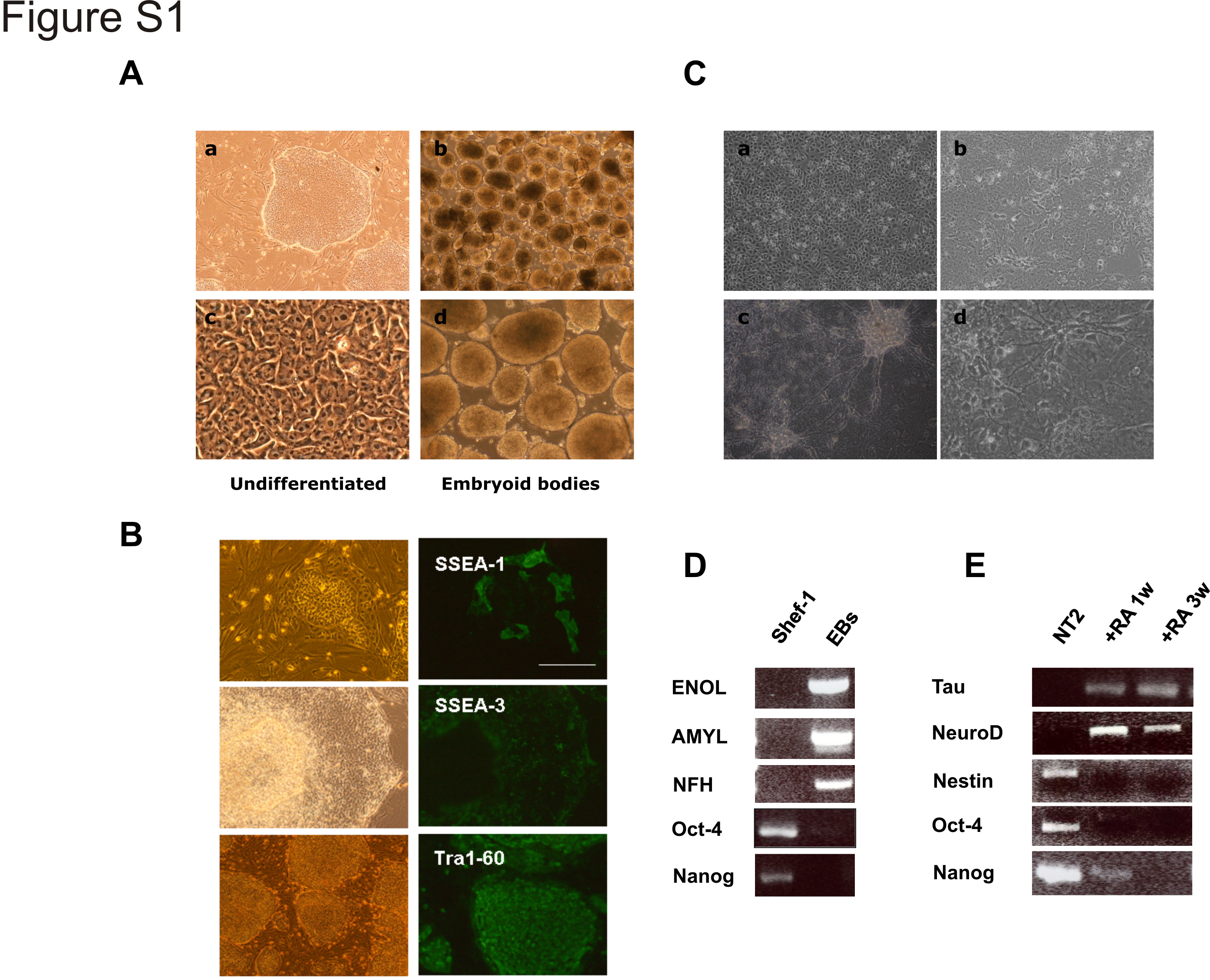

Supplement: Figure S1 — Culture and characterization of human embryonic stem cell line Shef-1 and the human carcinoma cell line NTera2 and differentiated cells. A) Culture of Shef-1 hES cell line and differentiation to embryoid bodyes (EBs). Shef-1 cells were grown in mouse embryonal fibroblast (MEFs) in gelatine-coated dishes in complete hESC medium with bFGF-2 (a). To differentiate to EBs (c), colonies were detached from MEFs and culture in suspension without bFGF-2 for 15 days. Panels b and d displayed hESC and EBs from Shef-1 cells respectively, at high magnification. B) Immunofluorescence analysis of specific cell-surface antigens, SSEA-1, SSEA-3 and TRA1-60 in Shef-1 hESC. Undifferentiated cells were strongly positive for SSEA-3 and TRA1-60 and only differentiated cells were stained by SSEA-1 antibodies. C) Induction of neuronal morphology in NT2 cell line. Undifferentiated cells (a) were treated with retinoic acid (RA) for 3–4 weeks. Samples were taken 1 week (b) and 3 weeks (c,d) and neuronal progenitors were observed. D) RT-PCR analysis of in vitro differentiated hESCs to EBs for detection of expressed genes of the three embryonic germ layers, enolase (mesoderm), amylase (endoderm) and neurofilament (ectoderm) and pluripitency genes (Nanog and Oct-4). E) RT-PCR analysis during the differentiation process of NT2 cell line to neuronal progenitors, Differentiated cells express well-characterized neuronal markers such as Tau or NeuroD whilst Nestin, a typical neuroectodermal marker, was downregulated by RA treatment. The transcription factors Nanog and Oct-4 were lost after 1 week in culture showing a right process of neural differentiation. (5.40 MB TIF) [file pone.0010192.s002.tif]

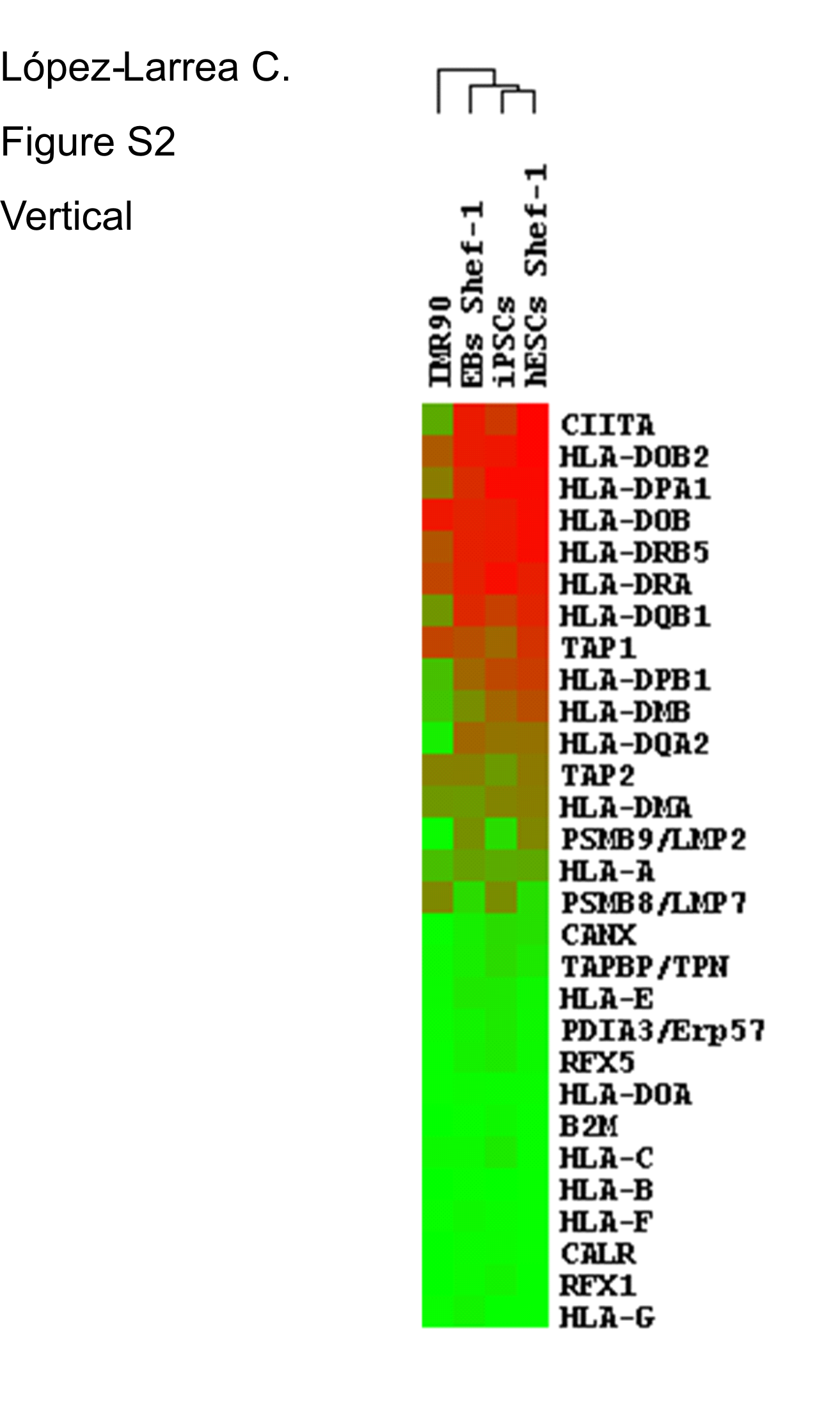

Supplement: Figure S2 — Methylation profiles of MHC genes and APM components. Methylation profiles of HLA class I, class II, antigen processing machinery (APM) genes and transcription factors (TF) involved in MHC regulation in hESCs, NT2 cell line, iPSCs and IMR90 fibroblast were obtained by Illumina arrays. Cluster analysis was based on correlation of methylation profiles of MHC and PM genes. The methylation levels vary from fully methylated (red) to fully unmethylated (green) sequences. (0.92 MB TIF) [file pone.0010192.s003.tif]
