# Supplementary material for: Epigenetic Mechanisms Regulate MHC and Antigen Processing Molecules in Human Embryonic and Induced Pluripotent Stem Cells
Source: PLoS One. 2010 Apr 16;5(4):e10192. doi: 10.1371/journal.pone.0010192 (PMC2855718; doi:10.1371/journal.pone.0010192)
Supplement: Table S2 — Primers used for real-time RT-PCR. (0.06 MB DOC) [file pone.0010192.s005.doc]

**Table S1.** **Primers for reverse-transcription PCR and real-time RT-PCR**

| **NAME** | **SEQUENCE** |
| --- | --- |
| **HLA-A s** | GCGGCTACTACAACCAGAGC |
| **HLA-A a** | CCAGGTAGGCTCTCAACTGC |
| **HLA-B s** | TCCTAGCAGTTGTGGTCATG |
| **HLA-B a** | TCAAGCTGTGAGAGACACAT |
| **HLA-C s** | TCCTGGTTGTCCTAGCTGTC |
| **HLA-C a** | CAGGCTTTACAAGTGATGAG |
| **HLA-E s** | CGCCTCCCCTATGTGTCTTA |
| **HLA-E a** | CGTGTTAGCCAGGATGGTTT |
| **HLA-F s** | CACAGAGCTTGTGGAGACCA |
| **HLA-F a** | GAGACTGCTCCCATCTCAGG |
| **HLA-G s** | CCACCACCCTGTCTTTGACT |
| **HLA-G a** | TGGCACGTGTATCTCTGCTC |
| **B2M s** | GTGCTCGCGCTACTCTCTCT |
| **B2M a** | TCAATGTCGGATGGATGAAA |
| **TAP-1 s** | ACGTCCACCCTGAGTGATTC |
| **TAP-1 a** | AGCTTTTCCCTAAACTTCTGGG |
| **TAP2 s** | ATCCCTCACTATTCTGGTCGT |
| **TAP2 a** | TGTAGGTGAAGCAGCCTCC |
| **TPN s** | TGGGTAAGGGACATCTGCTC |
| **TPN a** | ACCTGTCCTTGCAGGTATGG |
| **CNX s** | ATGGAAGGGAAGTGGTTGC |
| **CNX a** | TGGAAGCTTTGACTCCTTCAT |
| **CLR s** | GTGACGAGGAGAAAGATAAGG |
| **CLR a** | AACTCATCATCCTTGCAACG |
| **ERP57 s** | GCTAGAACTCACGGACGACA |
| **ERP57 a** | TCAGGGTTGGATATCCACTG |
| **LMP2 s** | CATCTACTGTGCACTCTCTG |
| **LMP2 a** | CAGCTGTAATAGTGACCAGG |
| **LMP7 s** | CAGACACAGACATGACAACC |
| **LMP7 a** | GCCACATGAGTGTCTTACTG |
| **DRA s** | AGACAAGTTCACCCCACCAG |
| **DRA a** | AGCATCAAACTCCCAGTGCT |
| **CIITA s** | CCGACACAGACACCATCAAC |
| **CIITA a** | TTTTCTGCCCAACTTCTGCT |
| **RFX5 s** | CTGATGCTAAGAGCCCCAAG |
| **RFX5 a** | TCAGTGTGCTCTTCCAGGTG |
| **GADPH s** | ACCACAGTCCATGCCATCAC |
| **GADPH a** | TCCACCACCCTGTTGCTGTA |
| **OCT4 s** | GACAACAATGAGAACCTTCAGGAGA |
| **OCT4 a** | CTGGCGCCGGTTACAGAACCA |
| **NANOG s** | GCTTGCCTTGCTTTGAAGCA |
| **NANOG a** | TTCTTGACTGGGACCTTGTC |
| **NESTIN s** | TCCAGGAACGGAAAATCAAG |
| **NESTIN a** | TAGAGACCTCCGTCGCTGTT |
| **TAU s** | GTAAAAGCAAAGACGGGACTGG |
| **TAU a** | CTCATTAGGCAACATCCATCAT |
| **NFH s** | TGAACACAGACGCTATGCGCTCAG |
| **NFH a** | CACCTTTATGTGAGTGGACACAGAG |
| **ENOL s** | TTCAAGTCGCCTGATGATCCC |
| **ENOL a** | TGCGTCCAGCAAAGATTGCCTTGTC |
| **AMYL s** | GCTGGGCTCAGTATTCCCCAAATAC |
| **AMYL a** | GACGACAATCTCTGACCTGAGTAGC |
