# Supplementary material for: Epigenetic Mechanisms Regulate MHC and Antigen Processing Molecules in Human Embryonic and Induced Pluripotent Stem Cells
Source: PLoS One. 2010 Apr 16;5(4):e10192. doi: 10.1371/journal.pone.0010192 (PMC2855718; doi:10.1371/journal.pone.0010192)
Supplement: Table S3 — Primers used for bisulfite sequencing. (0.04 MB DOC) [file pone.0010192.s006.doc]

**Table S2. Primers for bisulfite sequentiation PCR**

| **NAME** | **SEQUENCE** |
| --- | --- |
| **Bs-HLA-B s** | TTGTGTYGGGTTTTTTTTTTA |
| **Bs-HLA-B a** | ACAAAAACCATTTCCCTCC |
| **Bs-B2M s** | GAGATTTTAAGAAAAGGAAATTGA |
| **Bs-B2M a** | CCACCAAAAAAAACTTAAAAAA |
| **Bs-HLA-E s** | GAAATTTAAAAGTTTAAGAATTGTTGA |
| **Bs-HLA-E a** | TAAAAAAAAAATTCCATCTACCA |
| **Bs-HLA-F s** | GTGTTAGGTTTTTTTTTTTGGA |
| **Bs-HLA-F a** | CACTCCTCCCCACAAAAA |
| **Bs-HLA-G s** | TGGGTTAAGATTTAGGGAGATA |
| **Bs-HLA-G a** | TAACTTCTCTAAAAACCTATCACCTAA |
| **Bs-TAP-1 s** | TTTGGGGTATTGGTTTTTAA |
| **Bs-TAP-1 a** | CAACCCTAATACCCAATTTTC |
| **Bs-TAP-2 s** | TTTAGAATGAAGGTTTTGGTTG |
| **Bs-TAP-2 a** | TCCTATCRCCRAATACAA |
| **Bs-TPN s** | AGTATATATAGGAGTTGGGGGG |
| **Bs-TPN a** | TCAACCATAAAACCTCCTCTT |
| **Bs-LMP2 s** | TTGTTTYGGGGTAGATTTGTTT |
| **Bs-LMP2 a** | ACCAACCCTCAAACCCAA |
| **Bs-LMP7 s** | TTTTTAAGTTTTTTAATTTTTATGAGG |
| **Bs-LMP7 a** | ACTATTCTAAAATTTCCCCTCC |
| **Bs-Erp57 s** | GTTTYGGTTTATTTYGGGAT |
| **Bs-Erp57 a** | ACAAAAAACAACRCCACA |
| **Bs-DRA s** | TTGTTTAGAAGTTAGATTGGGG |
| **Bs-DRA a** | AATCCTAACACAAAAACTCCAC |
| **Bs-DRB s** | GAGGGGGGTTATAGTTTTTTT |
| **Bs-DRB a** | ACAAAAACCCCTTACACAAAT |
| **Bs-RFX5 s** | GAAATATAAAAGGGGAGGGTAAA |
| **Bs-RFX5 a** | TCTATCATTAATCACTACTACCACCC |
| **Bs-CIITA s** | GGTTTTATTTTGTAGAAGGTGG |
| **Bs-CIITA a** | AAAACACACAACCTCATCACT |
| **Bs-CIITA 1s** | ATTTTGTAGGGAGAGTTTTTTT |
| **Bs-CIITA 2a** | TCTAACAAATAAAACCCAACA |
