# Supplementary material for: Epigenetic Mechanisms Regulate MHC and Antigen Processing Molecules in Human Embryonic and Induced Pluripotent Stem Cells
Source: PLoS One. 2010 Apr 16;5(4):e10192. doi: 10.1371/journal.pone.0010192 (PMC2855718; doi:10.1371/journal.pone.0010192)
Supplement: Table S4 — Primers used for Chromatin Immunoprecipitation (ChIP) assay. (0.04 MB DOC) [file pone.0010192.s007.doc]

**Table S3.** **Primers for ChIP (chromatin immunuprecipitation) real-time RT- PCR.**

| **NAME** | **SEQUENCE** |
| --- | --- |
|  |  |
| **HLA-B s** | CTTGTGTCGGGTCCTTCTTC |
| **HLA-B a** | GCGTGGGGACTTTAGAACTG |
| **B2M s** | GAGGCTATCCAGCGTGAGTC |
| **B2M a** | GAAGTCACGGAGCGAGAGAG |
| **TAP1 s** | AACTGGTGCAAGTGGAAAGG |
| **TAP1 a** | ATCTGAGAATCTCGGGAGCA |
| **TAP2 s** | CAGCGCTGAAGCAGAAGTC |
| **TAP2 a** | AGGAGCGTGGAGTGGGTAGT |
| **TPN s** | GGCACCTTCACCTAACCAGA |
| **TPN a** | TGAAGCCTCCTCTTCCTCCT |
| **DRA s** | AAGAACCCTTCCCCTAGCAA |
| **DRA a** | GGGAGTGAGGCAGAACAGAC |
| **CIITA s** | AGGGCTTAAGGGAGTGTGGT |
| **CIITA a** | CAAGCTAAGCCAACATGCAA |
| **RFX5 s** | AACCGACCCCAGTTTTAACC |
| **RFX5 a** | GGTCAATAGGGGAGGGAAAA |
| **HLA-E s** | ACTGCTGATTGCTGGGAAAC |
| **HLA-E a** | CACCCATTGGGAATGAGAAC |
| **HLA-F s** | CCCATTTCTCACTCCCATTG |
| **HLA-F a** | TGCGTGGGACTTTAGAACCT |
| **HLA-G s** | ACGCTTGGCACAAGAGTAGC |
| **HLA-G a** | CCCGACACAGGTTAGGAGAA |
